# Time Series Classification for Predicting Biped Robot Step Viability

**DOI:** 10.3390/s24227107

**Published:** 2024-11-05

**Authors:** Jorge Igual, Pedro Parik-Americano, Eric Cito Becman, Arturo Forner-Cordero

**Affiliations:** 1 Instituto de Telecomunicaciones y Aplicaciones Multimedia (ITEAM), Departamento de Comunicaciones, Universitat Politècnica de València, 46022 Valencia, Spain; 2 Biomechatronics Laboratory, Mechatronics Department, Escola Politécnica, University of São Paulo (EP-USP), São Paulo 05508-220, Brazil; pedro.americano@usp.br (P.P.-A.); eric.becman@usp.br (E.C.B.); aforner@usp.br (A.F.-C.)

**Keywords:** biped stability, classification, robotics, machine learning, predicted step viability

## Abstract

The prediction of the stability of future steps taken by a biped robot is a very important task, since it allows the robot controller to adopt the necessary measures in order to minimize damages if a fall is predicted. We present a classifier to predict the viability of a given planned step taken by a biped robot, i.e., if it will be stable or unstable. The features of the classifier are extracted from a feature engineering process exploiting the useful information contained in the time series generated in the trajectory planning of the step. In order to state the problem as a supervised classification one, we need the ground truth class for each planned step. This is obtained using the Predicted Step Viability (PSV) criterion. We also present a procedure to obtain a balanced and challenging training/testing dataset of planned steps that contains many steps in the border between stable and non stable regions. Following this trajectory planning strategy for the creation of the dataset we are able to improve the robustness of the classifier. Results show that the classifier is able to obtain a 95% of ROC AUC for this demanding dataset using only four time series among all the signals required by PSV to check viability. This allows to replace the PSV stability criterion, which is safe, robust but impossible to apply in real-time, by a simple, fast and embeddable classifier that can run in real time consuming much less resources than the PSV.

## 1. Introduction

The challenge of maintaining step stability—the robot’s ability to stay balanced while fully executing a step—is critical not only for preventing falls and corresponding damages in robots, but also for ensuring smooth and energy-efficient locomotion [1,2,3]. Small deviations in trajectory planning, joint control, or external disturbances, such as uneven terrain, can lead to a loss of balance [4,5]. Traditional control approaches rely heavily on real-time feedback, in our case integrating sensors that monitor the robot’s position, orientation, and contact with the ground. However, these methods often fail to predict instabilities in advance, responding reactively rather than proactively to disturbances.

Research has increasingly focused on predictive methods for assessing and ensuring step stability. One of them is the *predicted step viability criterion* (PSV), which provides a solution for predicting whether a robot’s next step will result in stable or unstable locomotion. The criterion is based on the idea that if the current step leads to a configuration such that the capture point (the point associated with a complete stop where the energy in the system would not be enough to traverse the vertical position, remaining totally vertical) can be reduced in a future step, then it can, in the absence of disturbances, indefinitely be reduced in each subsequent step, thus reaching a full stop; i.e., any step that reduces the capture point in the next step results in a stable trajectory [6]. As a result of the robot configuration state, the time series of key variables, such as joint angles, velocities, swing leg position and center of mass displacement, are obtained and used to calculate the capture point and corresponding PSV criterion.

The use of machine learning has proven particularly effective in capturing the nonlinearity and complexity of bipedal movement [7,8,9]. Time series derived from motor primitives such as popular Dynamic Movement Primitives [10] allow for the analysis of dynamic, real-time behavior in a way that static measurements cannot [11]. By applying machine learning algorithms to these time series data, we can classify the viability of a step as either stable or unstable, based on past patterns and trends in the data [12,13]. In [14], a set of different machine learning algorithms such as support vector machines (SVMs) and random forests, were applied successfully to classify step viability with data from the robot’s legs and feet. These solutions not only improve the robot’s ability to anticipate and avoid falls, but also enables more efficient movement, as it reduces the need for conservative, energy-intensive gait adjustments that traditional reactive control systems often require [15,16].

In this work, we will analyze the interaction between various factors, such as the timing of the step, the distribution of weight, and the speed of joint movements, to determine whether the upcoming step will be successful. Furthermore, time series classification offers a robust framework for integrating various sensors, from accelerometers to force sensors, into a comprehensive model that predicts the outcome of each step with high accuracy.

In particular, we aim to further explore the classification of time series data in the context of bipedal locomotion, specifically focusing on the viability of steps in a biped robot under the PSV framework. The goal is to substitute the PSV by a simple and embeddable classifier. We will show that by analyzing the trajectory planning variables in the form of time series data, it is possible to anticipate whether a given step will be stable before it is executed, thus allowing the robot to make necessary adjustments to its trajectory or to adopt protection measures in case that the fall is inevitable.

The major advantage of our proposed solution is that the highly demanding in time and computational resources PSV criterion can be substituted by a classifier based on the extracted features of the different relevant time series. We will use the solutions obtained offline by the PSV criterion as the ground truth to train the classifier. By employing a range of feature engineering techniques, we aim to improve the accuracy of step stability predictions and provide new insights into the dynamic factors that influence successful bipedal walking. The results of this research will contribute to the development of more reliable and adaptive biped robots, capable of navigating complex environments with greater stability and efficiency.

## 2. Materials and Methods

In this section, we summarize the different methods to measure the stability of a biped gait and the one we use to generate the ground truth class for our dataset. We also explain how the generation of this dataset is designed in order to obtain a challenging and rich one. We present the classifier to be trained, focusing on how the desired operational characteristics of the classifier can be translated into restrictions in the form and size of the feature vector.

### 2.1. Stability Criteria

One of the most challenging problems faced by biped robots is ensuring stability during walking, especially in dynamic and unpredictable environments. Achieving stable locomotion requires careful planning of the robot’s trajectory and predicting whether each step will be stable [17]. Various methods have been proposed to evaluate the stability of biped robots, with the Zero Moment Point (ZMP) criterion being one of the most commonly used [18,19]. The ZMP is the point on the ground where the resulting torque of inertial and gravitational forces on the robot has no horizontal component. This requires the resulting forces between the feet and the ground to be located within the region defined by the contact between the feet and the ground. As a consequence, ZMP results in a high energy consumption with a slow and unnatural motion of the robot compared to human walking. The Foot Rotation Indicator (FRI) point has also been explored as an alternative for postural stability analysis [1]. These criteria ensure that the robot maintains balance, particularly during single-support phases, where stability is more susceptible to disturbances. Further advancements in the design of actuators and robotic joints, including hybrid serial-parallel legs and biomimetic designs, have helped reduce the energy demands and improve the dynamic responses of biped robots [20]. By incorporating flexible elements and reducing the inertia of the leg joints, these innovations allow biped robots to walk more efficiently while minimizing the risk of instability during transitions between steps.

Other techniques aimed at guaranteeing global stability of the biped during the gait cycle, such as the Limit Cycle approach [21]. Others are based on the capture point idea [2], that have been proven to be similar to the human gait experience [22,23]. An extension of the step capturability idea is the N-Step Capturability (N-SC) criterion that analyzes if the it is possible to reach the capture point in a certain number of steps [24], i.e., if there is a way to come to a stop in a given number of steps.

Inspired by this idea, the predicted step viability (PSV) was proposed [6]: a gait is considered stable if the biped is able to reach a capture point in a finite time, reducing the constraints imposed to the current step (it only has to end in a configuration that future steps are able to bring the robot to a capture point).

### 2.2. Predicted Step Viability

**Figure 1:**
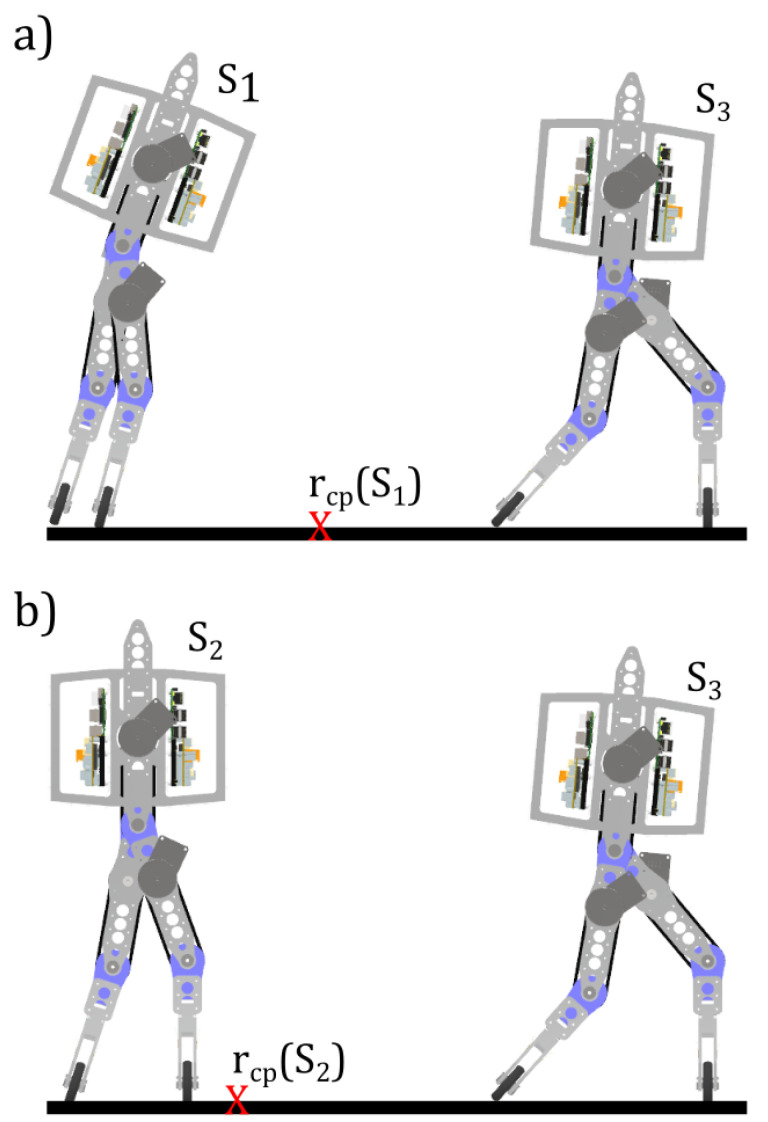
Illustration of the PSV concept in 2 different steps: (**a**) Capture point is initially distant from the robot’s support foot, giving the system less time to perform the step that brings the system to configuration $S_{3}$; (**b**) Capture point is initially closer to the feet, meaning the system has more time to perform the same step. If there is a set of actuations $U_{1}$ that make step a possible, then there is a set of actuations $U_{2}$ that make step b possible.

The concept of the PSV is illustrated in Figure 1. Let an underactuated robot with point feet be, at the beginning of the step, in a given set of initial conditions (positions and velocities) denoted $S_{1}$, with an initial capture point $r_{cp}\left(S_{1}\right)$. Due to the exponential divergence of the passive joint on the support foot, the robot has finite time to perform a step, denoted $T_{1}$. The exponential divergence rate is dictated, in the absence of actuations and external disturbances, by the position of the capture point: the farther away from the robot, the less time the system has before a fall.

Moreover, the only way to prevent a fall is to perform a step. If there exists a step that manages to drive the robot from configuration $S_{1}$ to $S_{3}$ respecting the systems constraints and reducing the distance to the capture point in the process, then in the next step, the system’s divergence is slower, resulting in time $T_{2}>T_{1}$ to perform the step that moves the robot from configuration $S_{2}$ to $S_{3}$. Since there existed a step that respected the systems constraints with less time and that managed to reduce the capture point distance, then there exists a next step that can achieve the same in more time. Thus, once a viable step is found that reduces the capture point distance, in the absence of disturbances, all future steps will also remain viable. Conversely, if no step can reduce or maintain the capture point distance, instability and eventual failure to complete the gait are inevitable.

Due to the multistage optimization nature of the problem, the PSV not only minimizes motor torques and ensures stability, but also allows for the planning and execution of non-cyclic gait, similar to a human walking on uneven surfaces. With simple desired parameters like step length and desired ranged of trunk motion, the algorithm is capable of adjusting step duration, foot contact timing, and other variables, mimicking human gait’s ability to recover from disturbances, such as tripping.

Despite its capabilities, the major limitation of PSV is its computational complexity. The algorithm’s reliance on full robot dynamics to assess the stability of each possible step prevents real-time application on embedded systems, especially in fast-paced, dynamic environments. In its current form, PSV cannot be employed directly for real-time control of biped robots or exoskeletons, limiting its usefulness in practical scenarios. In this work, we substitute it by a classifier trained on PSV data.

### 2.3. Dataset Generation

Data quality is paramount for the effectiveness of any machine learning classifier [25]. In our case, constructing the dataset involves adhering to a few important requirements and constraints. First, to prevent bias toward one class, it is essential to balance the dataset, ensuring that the number of stable and unstable planned steps is approximately equal. This avoids the need for preprocessing techniques, such as SMOTE, which would otherwise generate interpolated steps to artificially balance the dataset. In this context, SMOTE is impractical because it would require simulating the entire time series for each variable representing a step.

Second, the calculated step trajectories must accurately reflect the real dynamics of bipedal gait. Simplified models, such as the inverted pendulum, while useful for reducing computational complexity and having been successfully applied in some real world scenarios, do not capture the full range of behavior observed in real-world gait. These models introduce increasing error as the robot moves away from ideal configurations, making them inadequate for predicting recoverability or planning, particularly after large disturbances like trips or pushes. Therefore, a more complex, realistic model is necessary to generate data that is both precise and relevant for real-world applications.

**Figure 2:**
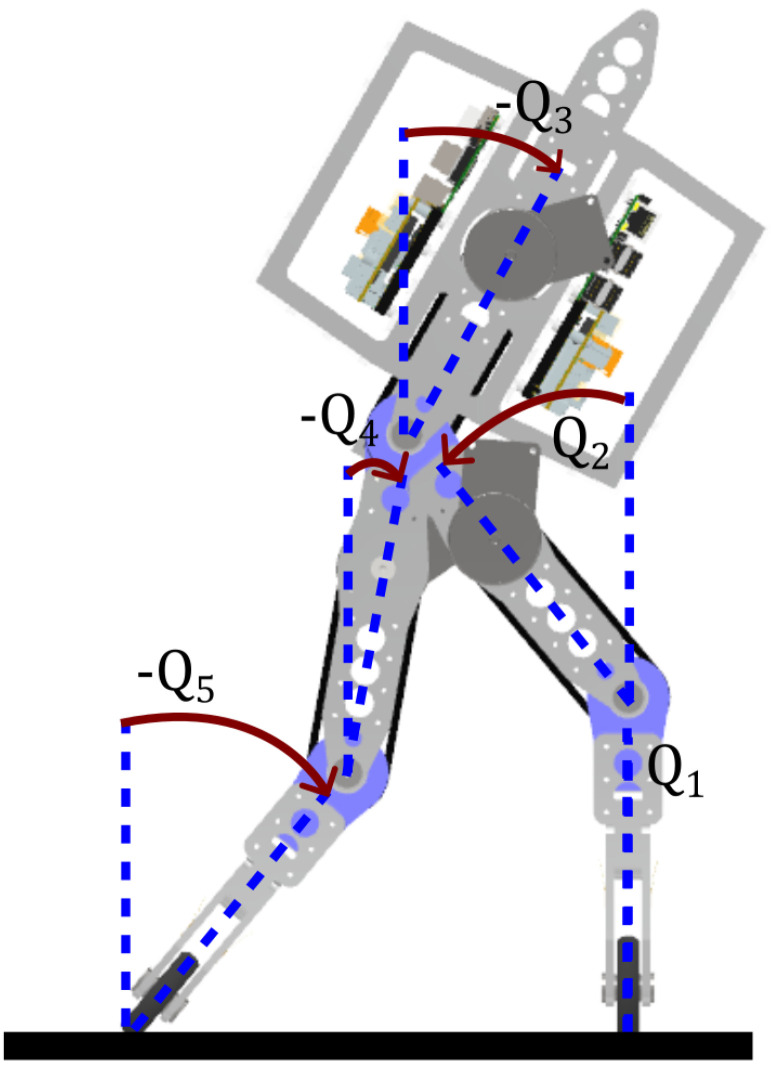
Robotic model for the dataset generation.

To satisfy these requirements, we employed the PSV method, sampled at 1 KHz, to generate a large dataset, exploring a broad range of initial conditions while maintaining balance between stable and unstable steps. The robotic model used in this dataset construction is a modified version of the classic 5-segment, planar robot with point feet, RABBIT [26]. It is a modular, symmetrical robot, with most of its mass concentrated in the trunk, where the electronics are housed, making the trunk’s dynamics critical to the system’s center of mass (CoM), see Table 1. The angles are defined relative to the vertical, and sign follows the standard trigonometric phasor convention, as shown in Figure 2. Since it is a planar robot, its motion is confined to the sagittal plane, with inter-part collisions disabled.

To generate the set of possible initial conditions, three primary constraints were imposed to reflect expected configurations following a step. First, the robot is assumed to be moving forward, meaning the support foot is placed in front of the swing foot and the trunk is not initially bent backward. Additionally, all joint velocities are initially negative, indicating forward motion. Second, the robot’s legs are subject to mechanical limits resembling human joints, where the internal angle between the thigh and leg must remain less than π, preventing forward knee bending. Third, because the initial configuration occurs immediately after the swing foot contacts the ground, both feet are initially in contact with the ground, but the new swing foot must immediately leave the ground, meaning there is no double stance phase except during impact.

These 3 constraints can be represented by the following 5 equations:

$Q_{1}\in ]-\left(\pi /2\right);0]$ and ${\dot{Q}}_{1}<0$;
$Q_{2}\in ]q_{1};\pi +Q_{1}]$ and ${\dot{Q}}_{2}<0$;
$Q_{3}\in ]-\left(\pi /2\right);0]$ and ${\dot{Q}}_{3}<0$;
$Q_{4}\in ]\arccos\left(\frac{l_{1}\cos\left(Q_{1}\right)+l_{2}\cos\left(Q_{2}\right)}{l_{1}+l_{2}}\right);Q_{2}]$ and ${\dot{Q}}_{4}<0$;
$Q_{5}=\arccos\left(\frac{l_{1}\cos\left(Q_{1}\right)+l_{2}\cos\left(Q_{2}\right)-l_{2}\cos\left(Q_{4}\right)}{l_{1}}\right)$ and ${\dot{Q}}_{5}<0$.

To ensure a balance between successful and failed steps, three additional filters were applied to the resulting workspace. This approach not only ensures a balanced dataset, but also focuses on conditions near the boundary between stability and instability, which are the most challenging and critical for accurate identification of stable and unstable configurations. First, the robot’s initial CoM height must be above 74% of the average CoM height in healthy human gait, scaled to the robot’s dimensions. This prevents configurations too far from steady-state walking. Second, the initial vertical velocity of the CoM must be greater than −0.6 m/s to avoid scenarios where the robot is driven too forcefully into the ground, which could be irrecoverable due to the passive dynamics of the support foot joint. Third, we compute the theoretical n-step capturability of each configuration, and exclude cases where more than three steps would be required to reach the capture point. This is because, even with the earlier constraints, most initial conditions fall outside the idealized inverted pendulum model, and the simplified model would misclassify steps requiring more than three recoverable steps.

With these rules in place, and using a grid resolution of 0.3140 radians for positions and 3 rad/s for velocities, we simulated 74,618 different conditions, and the optimal trajectory was planned using the PSV criterion. From these, 44.4% were classified as stable, thereby meeting the dataset balance requirement and validating our strategy for generating representative future datasets. From this pool, we selected 4000 stable and 4000 unstable steps, ensuring uniform coverage across the variable space to include all regions of interest. This selection also reduces overfitting, as the model is trained on a subset of all possible conditions, enhancing its robustness when exposed to new, unseen data.

It is also important to remember that the PSV uses a complete model of the robot instead of a simplified one such as the inverted pendulum to perform the multi-stage optimization. This means that the result obtained by the PSV is the ground truth under ideal circumstances (no obstacles, no noise, no other changes not included in the model). The use of the PSV to obtain the ground truth class of the step and the planned trajectory for the set of variables during the step has the advantage of obtaining a dataset of great quality for the training process. The disadvantage is that it takes a lot of time to compute all these calculations. These conditions were ran in a Linux virtual machine in 3 different computers, and each step took up to 3 min of calculation to reach the optimal trajectory planning.

For each simulated step, we recorded time series data of positions, velocities, and torques for the five segments shown in Figure 2, as well as the corresponding class label (stable or unstable). Notably, since the PSV criterion requires both the current and next step for multistage optimization, the time series for the second step is only used to calculate the capture point and assess the stability of the current step, as defined by the PSV stability criterion. With these 15 features, additional time series can be derived, such as the CoM positions and velocities for each joint, as well as for the entire robot. We also capture the end-effector (foot) position of the swing leg and the capture point for further analysis.

### 2.4. Classifier

The PSV criterion cannot be implemented in robots in real time. However, it can be used to obtain the ground truth of many different robot configurations and planned trajectories in order to train a machine learning based classifier to detect if the step associated with those trajectories will be stable or not. In [14], different classifiers were trained and tested to classify the initial conditions of the step. In that case, the random forest classifier obtained the best results taking into consideration not only the accuracy, but also the computational and time cost. But the use of only the initial condition of the robot’s next step does not exploit the dynamics of the corresponding time series captured during the step.

On the other hand, the use of all robot configuration variables in obtaining a classifier has the disadvantage that the feature space can be increased unnecessarily, since some of these variables are related and not all of them have the same importance from the stability perspective. For example, the vertical coordinate of the center of mass can be calculated from other variables, and it is clearly more intuitively related to the stability of the biped than other variables.

In this work, we use all the information encoded in the time series in order to obtain a low cost time series classifier with few hyperparameters so it can be deployed easily in real case scenarios. At the same time, we want to obtain an intuitive feature space, so instead of using multiple related variables we will keep the interest in the most important ones from a robotic stability point of view.

### 2.5. Model Restrictions: Time Series Selection

To build a classifier to predict the stability of next steps is a machine learning problem that can be solved in many different ways. The two most typical approaches are the instance-based and feature-based solutions. In the former case, the class assigned to the new time series is the one of the most similar time series (distances are calculated in the time domain vectors) while, in the latter case, the time series are encoded in a feature vector (distances and corresponding classification is carried out in the feature space). Since we are dealing with many time series of different durations, we will follow the feature-based approach.

In order to define the feature space, in addition to a good accuracy of the corresponding model, we are also interested in obtaining a classifier that has the following characteristics:

- Applicability (usability): If the implementation is too complicated and requires a lot or resources (time, memory, energy), it is not an acceptable solution. This is what happens with PSV criterion, while theoretically solve the stability prediction problem, it can not be used in practice. This fact also limits the use of deep learning approaches, since we do not have a huge amount of data and the neural network architecture should not be very complicated either.
- Interpretability: It must be interpretable, i.e., we need to provide a clear connection between the performance of the model and the variables that are commonly used to understand the stability of a biped robot. This is the same that providing a classifier that can be interpretable in terms of intuitive human gait experience. For example, a model that is able to predict that the robot will fall because the angle $Q_{3}$ between the trunk and the vertical is too large is preferable to other more sophisticated, but less intuitive, models. In summary, the most data comprehensive the classifier is, the more likelihood that it could be accepted in the robot industrial community.
- Fast: it must be able to work in real time.
- Simple: It is preferable if it can work with few variables. Related to previous property, considering a real robot case with different sensors and actuators, we want to limit the dependency of the classifier from failures of any of these sensors as much as possible, so the predictor can work in hardware failure situations. When training the classifier, we can use the relative importance of each feature in the predictive power of the model to reduce the dimension of the classifier, speeding up its calculations and reducing the amount of consumed resources during deployment in real robots. If the sensors that fail are the ones that we are using, we can always expand the classifier to include other variables with the obvious corresponding performance reduction.

Attending to all these restrictions, we start by defining a minimum number of four time series to be used in the classifier. They are obtained from the raw data captured by the sensors (position and velocity for each joint of the robot), and satisfy the condition that they must be gait descriptors easily understandable by humans so its predictive power can be explained in a simple intuitive way. For example, if the trunk inclination is very large and negative (back leaning during the step), it is straightforward to predict that the robot will fall backwards. The three chosen variables are:

- The trunk inclination given by the angle $Q_{3}$ in Figure 2.
- The height of the global center of mass represented by the variable $CoM_{y}$.
- The coordinates of the swing leg, denoted by $\left(PSW_{x},PSW_{y}\right)$.

### 2.6. Feature Engineering

Once the time series of interest are selected, we must obtain the feature vector to be used in the classifier. Because an important requirement in the design of the classifier is its future implementation in real robots, we have to define a set of features as informative as possible in a low-dimensional feature space. In this way we also minimize the risk of overfitting. We will consider three different feature engineering approaches that focus on the properties of the signals from different perspectives. The only restriction is that the dimension of the feature space is the same in all three domains.

#### 2.6.1. Statistical Features

We refer to statistical features to those that summarize the distribution of values of the given time series, e.g., we do not exploit any temporal dependency between samples (the results will be the same if the time series data are shuffled). They provide a general idea about the distribution of values during the time series, encoding information about the values distribution such as the central value, dispersion, and shape of the distribution.

The set of statistical features that we calculate for the time series of the corresponding variable x(t) of duration *N* samples, $x_{1},x_{2},\ldots ,x_{N}$, in each robot’s step are:

- Mean (μ):
  (1) $$\mu =\frac{1}{N}\sum_{i=1}^{N}x_{i}$$
- Variance ($\sigma^{2}$):
  (2) $$\sigma^{2}=\frac{1}{N}\sum_{i=1}^{N}{\left(x_{i}-\mu \right)}^{2}$$
- Skewness ($\kappa_{3}$):
  (3) $$\kappa_{3}=\frac{\frac{1}{N}\sum_{i=1}^{N}{\left(x_{i}-\mu \right)}^{3}}{{\left(\sqrt{\frac{1}{N}\sum_{i=1}^{N}{\left(x_{i}-\mu \right)}^{2}}\right)}^{3}}$$
- Kurtosis ($\kappa_{4}$):
  (4) $$\kappa_{4}=\frac{\frac{1}{N}\sum_{i=1}^{N}{\left(x_{i}-\mu \right)}^{4}}{{\left(\frac{1}{N}\sum_{i=1}^{N}{\left(x_{i}-\mu \right)}^{2}\right)}^{2}}$$
- Minimum (min):
  (5) $$\min\left(x\right)=\min\left\{x_{1},x_{2},\ldots ,x_{N}\right\}$$
- Maximum (max):
  (6) $$\max\left(x\right)=\max\left\{x_{1},x_{2},\ldots ,x_{N}\right\}$$
- 10% quantile ($q_{10}$):
  (7) $$q_{10}=x_{\left\lceil 0.1\cdot (N-1)+1\right\rceil }^{\prime }\;\mathrm{where}\;\left\{x_{1}^{\prime },x_{2}^{\prime },\ldots ,x_{N}^{\prime }\right\}=\mathrm{sort}\left\{x_{1},x_{2},\ldots ,x_{N}\right\}$$

The minimum and maximum establish the limit values while the 10% quantile $q_{10}$ is the value where the 90% of the values are greater than it. The first four order central normalized moments provide different information about the shape of the distribution: the mean μ represents the average value of the time series, the variance $\sigma^{2}$ represents how spread the values are, the skewness $k_{3}$ represents the asymmetry of the distribution around the mean value ($k_{3}>0$ when extreme data values are greater and $k_{3}<0$ when extreme values are smaller), and kurtosis $k_{4}$ represents the flatness of the distribution ($k_{4}=0$ for Gaussian, $k_{4}>0$ for tailored distributions such as Laplacian, and $k_{4}<0$ for flatter ones such as the uniform random variable). For example, if an stability indicator is that the vertical coordinate of the center of mass is relatively stable during the time series, it should translate to a mean value around the height of the hip, the variance should be small since the $CoM_{y}$ time series follow a parabollic movement, and the skewness should be close to zero, since it should be symmetric around the average height.

#### 2.6.2. Temporal Features

By temporal features, we refer to those that try to capture the dynamic aspects of the time series over time. They focus on the patterns and changes over time, rather than just summarizing the overall characteristics of the data as the previous statistical features did (note that most of the statistical features as we defined in previous equations can be consider temporal features, since they involve time averages, but we prefer to group them as statistical features to make clear when the temporal structure is used or not).

The temporal features we use are:

- Length (*N*): duration of the time series in samples, i.e., the duration of the step.
- Number of peaks with support two, i.e., it is considered a peak if it is higher than both its two preceding and two succeeding neighbors ($P_{2}$):
  (8) $$P_{2}=\sum_{i=3}^{N-2}I\left(x_{i}>\max\left(x_{i-2},x_{i-1},x_{i+1},x_{i+2}\right)\right)$$
  where I(·) is the indicator function that returns 1 if the condition inside is true, and 0 otherwise.
- Number of crossings of the mean value ($C_{\mu }$): the number of times the time series crosses the mean value:
  (9) $$C_{\mu }=\sum_{i=1}^{N-1}I\left(\left(x_{i}>\mu \;\mathrm{and}\;x_{i+1}<\mu \right)\;\mathrm{or}\;\left(x_{i}<\mu \;\mathrm{and}\;x_{i+1}>\mu \right)\right)$$
- Maximum length of time series segments where consecutive samples are greater than the mean value ($L_{1}$):
  (10) $$L_{1}=\max\left\{\mathrm{length}\left(\{x_{i}|x_{i}>\mu \}\right)\right\}$$
- Maximum length of consecutive samples where the values are smaller than the mean value ($L_{2}$):
  (11) $$L_{2}=\max\left\{\mathrm{length}\left(\{x_{i}|x_{i}<\mu \}\right)\right\}$$
- Average of the absolute first differences of the time series ($\mu_{\Delta x}$):
  (12) $$\mu_{\Delta x}=\frac{1}{N-1}\sum_{i=1}^{N-1}\left|x_{i+1}-x_{i}\right|$$
- Average value over the autocorrelation function for different lags ($\hat{R_{l}}$):
  (13) $$\hat{R_{l}}=\frac{1}{L+1}\sum_{l=0}^{L}R\left(l\right)$$
  (14) $$R\left(l\right)=\frac{1}{N-l}\sum_{i=1}^{N-l}\left(x_{i}-\mu \right)\left(x_{i+l}-\mu \right)$$

#### 2.6.3. Advanced Features

The third set of features try to encapsulate the complexity and non linear behavior of the time series. Since the gait of a biped robot is plenty of nonlinear calculations, we expect that this feature set is able to capture the differences between a successful step and the ones not recoverable.

The advanced features are:

- Time Reversal Asymmetry Statistic (TRA(l)): Measures the asymmetry in the time series when reversed, which can indicate non-linear dynamics. There are different implementations of this idea. We use the following one and calculate two values l=1 and l=5:
  (15) $$TRA\left(l\right)=\frac{1}{N-2l}\sum_{i=1}^{N-2l}\left(x_{i+2l}^{2}\cdot x_{i+l}-x_{i}^{2}\cdot x_{i+l}\right)$$
- $C_{3}$ statistic ($C_{3}\left(l\right)$): A higher order autocovariance (a generalization of linear autocovariance introducing more than one lag to capture higher order dependencies). We use the values for l=1 and l=5.
  (16) $$C_{3}\left(l\right)=\frac{1}{\left(N-2l\right)}\sum_{n=1}^{N-2l}x\left[n\right]\cdot x\left[n+l\right]\cdot x\left[n+2l\right]$$
- Correlation dimension ($C_{d}$) measures the dimensionality of the space that the data occupies (low-dimensional chaotic systems or higher-dimensional stochastic processes). It is estimated as the slope of the log-log plot of the correlation integral C(ϵ) vs. ϵ:
  (17) $$C\left(\epsilon \right)=\epsilon^{C_{d}}$$
  where C(ϵ) is a measure of the probability that two points, selected randomly from the dataset, are within a distance ϵ:
  (18) $$C\left(\epsilon \right)=\frac{1}{N(N-1)}\sum_{i\neq j}I\left[∥X_{i}-X_{j}∥\leq \epsilon \right]$$
  and $X_{i}=\left(x_{i},x_{i+\tau },x_{i+2\tau },\ldots ,x_{i+(d-1)\tau }\right)$ is the vector in the reconstructed phase space using time delay embedding.
- Hurst exponent (*H*). This indicates if the time series is purely random, trending, or rather mean reverting (long term memory). It is estimated as the slope of the log-log plot of the range rescaled by the standard deviation vs. time:
  (19) $$R/S∼t^{H}$$
  with range and standard deviation calculated for several segments of changing duration *t*:
  (20) $$R\left(t\right)=\max_{1\leq i\leq t}\left(\sum_{j=1}^{i}\left(x_{j}-\mu \right)\right)-\min_{1\leq i\leq t}\left(\sum_{j=1}^{i}\left(x_{j}-\mu \right)\right)$$
  (21) $$S\left(t\right)=\sqrt{\frac{1}{t}\sum_{i=1}^{t}{\left(x_{i}-\mu \right)}^{2}}$$
  White noise has H=0.5, while time series with mean-reverting characteristics (increase in the value is likely to be followed by a decrease and vice versa, i.e., negative dependencies) have H<0.5 and H>0.5 for those that exhibit some positive dependency on previous values.
- Detrended Fluctuation Analysis (DFA) is another measure of long term dependencies that tries to avoid false correlations appearing in non stationary processes (unlike the Hurst exponent, which always indicates long-term correlations for any non-stationary process). Once again, we have to estimate a logarithmic relationship, in this case between the overall fluctuation function F(s) for different length segments *s*:
  (22) $$F\left(s\right)∼s^{\alpha }$$
  where F(s) is obtained averaging the fluctuation F(ν,s) over all $N_{s}=\left\lfloor \frac{N}{s}\right\rfloor$ segments:
  (23) $$F\left(s\right)=\sqrt{\frac{1}{N_{s}}\sum_{\nu =1}^{N_{s}}F{\left(\nu ,s\right)}^{2}}$$
  with F(ν,s) the root mean square fluctuation between the integrated time series (the profile) $Y\left(m\right)=\sum_{k=1}^{m}\left(x_{k}-\mu \right),\;m=1,2,\ldots ,N$, evaluated at point *m* in segment ν, and the fitted polynomial trend in that segment at the same point $P_{\nu }\left(m\right)$:
  (24) $$F\left(\nu ,s\right)=\sqrt{\frac{1}{s}\sum_{m=1}^{s}{\left(Y\left(\left(\nu -1\right)s+m\right)-P_{\nu }\left(m\right)\right)}^{2}}$$
  The value of the estimated exponent α is associated with the nature of the time series, e.g., α<0.5 for anti-correlated (large values are more likely to be followed by small values and vice versa) ones, α=0.5 for white noise (uncorrelated time series), 0.5<α<1 for long range correlations (long term memory), and α>1 for non-stationary time series with a trend.

### 2.7. Feature Importance

Since we are interested in obtaining an intuitive self explanatory relationship between the outcome of the predictor and the feature vector values, we need to rank the features based on its explanatory power. Because we are using a random forest classifier, we can measure how much a feature decreases the impurity (e.g., Gini index [27]) of a node, and average across all trees in the ensemble. This solution is called Mean Decrease in Impurity (MDI), and it basically reflects the average decrease in impurity (Gini) due to splits involving a particular feature across all trees. A feature that frequently appears in significant splits and leads to large reductions in impurity will have higher importance. To test the robustness of the MDI ranked features, we calculate another tree-based feature rank obtained by the Extra Trees algorithm [28], which adds randomness to the splitting points, since it randomly selects cut points for features at each tree split.

In order to assure that the results obtained with these methods are not model-dependent (ensemble of trees) and there is no bias towards some features [29,30], we calculate the importance of each feature using an alternative model-agnostic algorithm, the Random Feature Permutation [31]. In this case, after training the classifier, the values of a single feature are permuted to break its relationship with the class variable, and the decrease in model performance is measured; i.e., it shows the reduction in model accuracy when a feature is shuffled, indicating its importance.

The advantage of ranking the feature variables based on its predictive power is that we can reduce the dimensionality of the final model, obtaining a much simpler classifier with a similar performance that can help in the design of the controller of the robot.

An alternative way to reduce the dimension of the feature vector is to use a feature space transformation. The most popular one is the Principal Component Analysis [32], which transforms the original features into a new set of orthogonal components (principal components) that capture the maximum variance in the data, typically 90% or 95%. Note that PCA is a technique to reduce the feature vector before the training process. This means that we mix the input features so the interpretability of the resulting feature vector is more complicated than the methods that analyze the importance of the features after the training process, since they are not combining features. We will use the results obtained with the PCA transformation as a benchmark for comparison to the methods based on feature information. This PCA result will be helpful to test if the nonlinear processes involved in the planning of a robot step can be approximated by linear models, since the directions of maximum variance might not align with the most informative or meaningful structures in the time series data.

## 3. Results

In this Section we will study the performance of the classifiers from different points of view. First, we will compare the performance of the classifiers looking at the metrics obtained by each of them. Then, we will analyze which features are more relevant for each classifier and if it is possible to obtain a simple intuitive classifier in the different feature domains.

### 3.1. Performance Metrics

We trained three random forest classifiers, each one for the corresponding feature space: statistical, temporal and advanced features. We used standard K-fold crossvalidation with K=5. In Table 2, we show the balanced accuracy, precision, recall, F1 score, specificity, negative predictive value (NPV), ROC AUC and average precision obtained by them for the test data after training on the corresponding feature space (statistical, temporal, or advanced features). As a reference or baseline, we also include the results obtained by a classifier trained only with a single value, the initial condition of the step (right column in the table, Initial). As we can see, the three proposed classifiers exploiting the whole time series information obtain a much better performance than the reference one. This shows that the time series classifiers are able to capture a much better representation of each class and correspondingly a much better performance. The most interesting conclusion from these results is that all three proposed classifiers obtain a similar performance in all metrics, proving that the problem can be solved in different feature spaces and that the performance is similar for each type of error. In our case, a false positive (predicted stable step when it is not) is worse than a false negative, since it can produce a great damage to the robot when falling unexpectedly.

**Figure 3:**
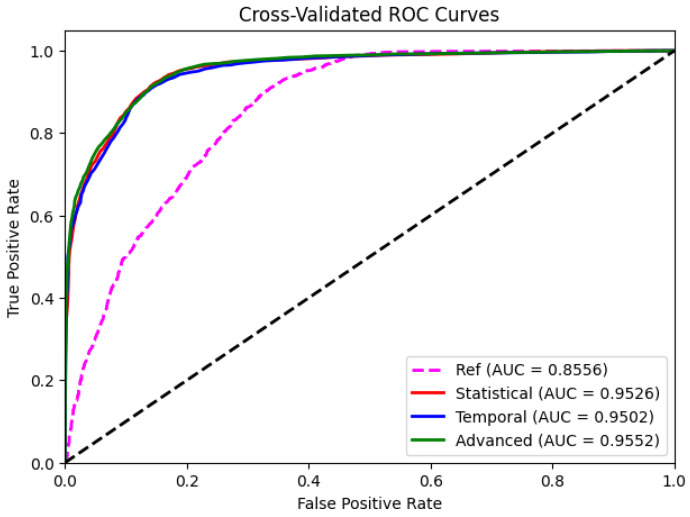
ROC curves for the time series classifiers using statistical (red), temporal (blue), and advanced (red) features, and the one obtained by the baseline classifier (magenta).

To verify it visually, we plot the corresponding ROC curves in Figure 3. The AUC ROC values are very similar no matter the nature of the features. The three classifiers achieve a 0.95 AUC, proving that they are able to distinguish between the stable and unstable steps. As we can see in the figure, this is not the case of the baseline classifier that does not use the dynamics of the step.

### 3.2. Feature Importance

Once we have shown that a classifier can be obtained to predict the stability of the step, the next goal is to obtain a simple classifier that can be implemented easily in a robot; i.e., once we have analyzed the good performance no matter the feature space where the classifier is trained, we look for if this is still true when reducing the number of features for each classifier.

**Figure 4:**
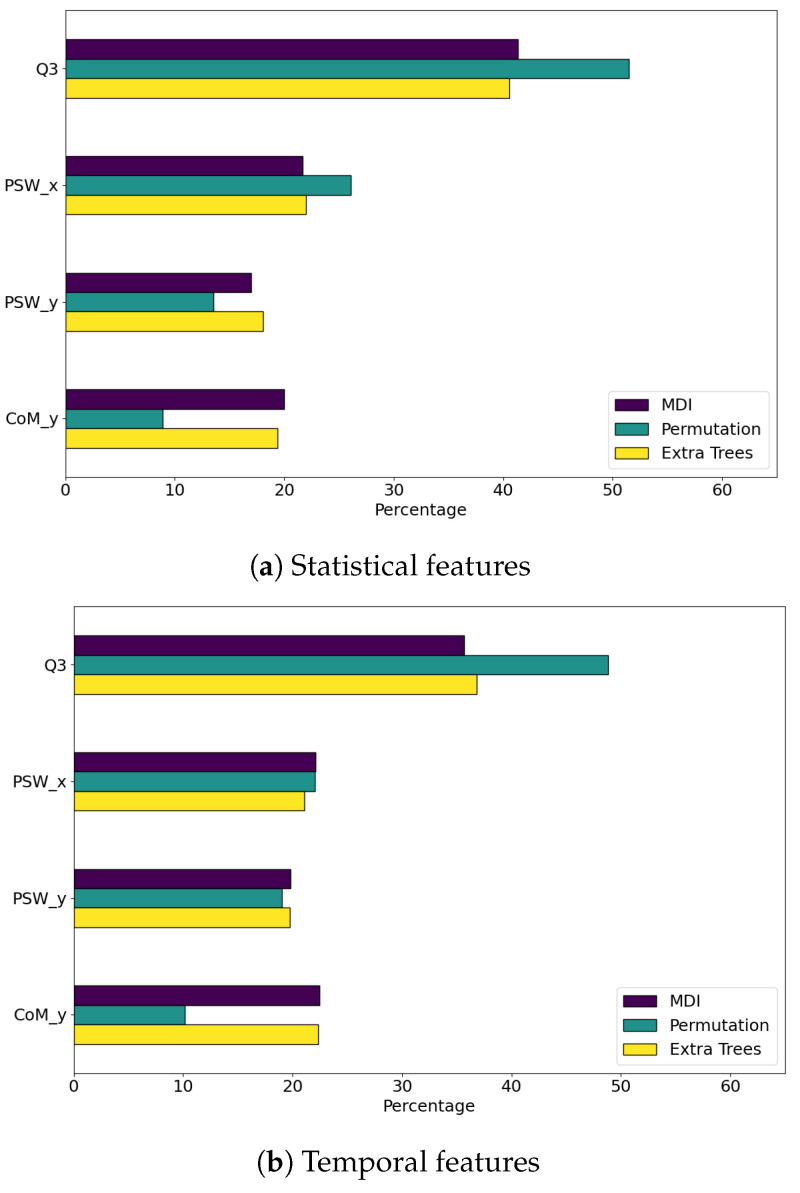

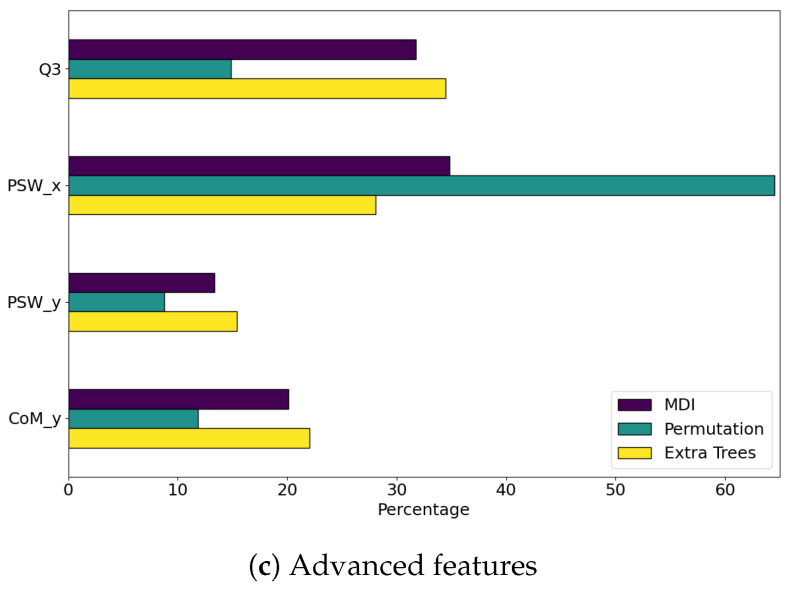
Aggregated feature importance as percentages by variable for statistical (**a**), temporal (**b**) and advanced (**c**) feature based classifiers.

We calculated the feature importance for each set of features using the three different methods explained in Section 2.7. In Figure 4, we obtained the accumulated feature importance for each variable and method. The figure shows that the time series that contain more information to discriminate if the step will be stable or not is the trunk inclination $Q_{3}$. In the case of using the classifier obtained with the advanced features, the horizontal location of the swing leg $PSW_{x}$ also becomes very important.

As we expected, both methods based on the tree feature ranking, MDI and Extra Trees, obtain close results for all classifiers. However, in the case of the Permutation method, there is a clear difference in the most relevant variable, depending on which characteristic of the time series we emphasize. When the classifier is trained on statistical or temporal features, $Q_{3}$ is by large the most interesting variable, while if the classifier is exploiting the advanced features of the time series, the $PSW_{x}$ variable is more informative.

**Figure 5:**
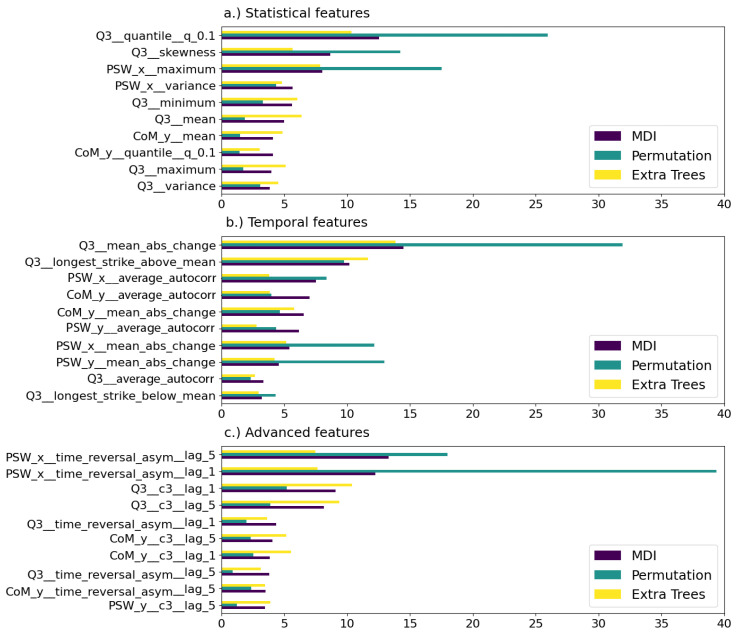
Top ten ranked features for each classifier according to MDI, permutation, and Extra Trees methods.

In Figure 5, we plot the top ten features for each method and set of features. After previous global analysis, it is not surprising that most of the top features are related to $Q_{3}$ and $PSW_{x}$ time series, no matter the domain we analyze.

### 3.3. Low Dimensional Classifiers

Once we know the most representative features for each domain, we can train new classifiers using only the small set of features for each family of features.

The results obtained using only the five top features for each method are summarized in Table 3. We also include, as a reference, the results obtained if the reduction of the dimension feature space is carried out by standard techniques such as PCA. We can use the results for the PCA case (we adjust the percentage of variance to be kept until we obtain the same feature space dimension) as a reference value. As we can see, all the new classifiers obtain a better performance than the PCA-based solution. This means that the standard variance based approach to reduce the dimensions of the classifier and, therefore, reduce the amount of resources can be improved by our approach, since we are interested in keeping the most informative features from a classification point of view instead of a variance point of view. This results show why it is important to exploit the nonlinearities of the system behind the biped gait control instead of general approaches as PCA more appropriate for second order correlation solutions.

In the figure and in the AUC ROC values, we can see that the performance of these new simple classifiers is not far from the results obtained when using all the set of initial features, i.e., it is possible to obtain a simple intuitive classifier in the different domains that only needs a five dimensional feature space mostly related to $Q_{3}$ and $PSW_{x}$ to substitute the costly PSV criterion in order to predict the stability of the step. This open the door to a practical implementations of the PSV in robots using as a proxy the trained classifiers.

**Figure 6:**
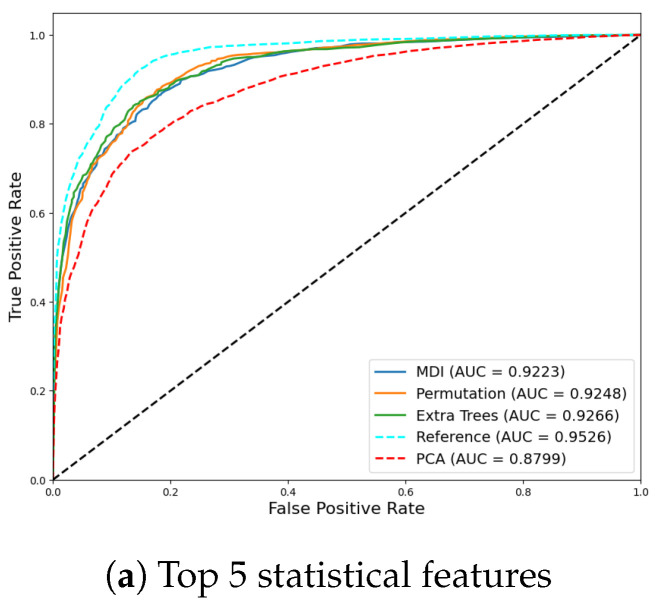

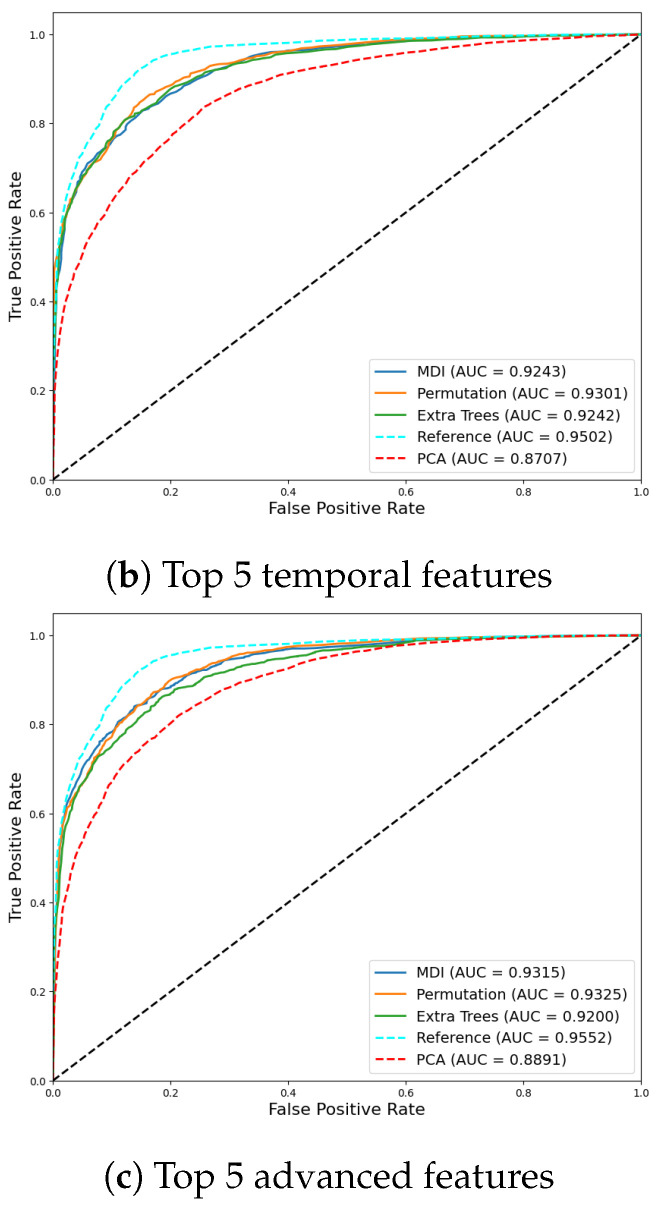
ROC curves for the classifiers using top 5 features according to MDI (blue), Permutation (orange), Extra Trees (green) and PCA (red) method for statistical (**a**), temporal (**b**), and advanced (**c**) features. In cyan is the ROC using all of the features.

To visualize the performance of the simplified classifiers, in Figure 6, we plot the ROC curves obtained for the statistical, temporal and advanced features, respectively. For comparison purposes, in each figure, we include the ROC for the PCA with five principal components and the ROC when using the whole set of corresponding features.

## 4. Conclusions

Guaranteeing robot stability is probably the most important issue when planning the joint trajectories of biped robots. Although it is possible to calculate a stable step using the PSV procedures, it is not useful for real time scenarios. It requires too many computational time, so alternative practical solutions are necessary. In this paper, we have shown how machine learning solutions can substitute the original PSV calculations while providing stable solutions.

After comparing the results obtained exploiting different kinds of information contained in the time series (statistical, temporal, and more advanced features), we have seen that any of the studied feature spaces is able to discriminate between stable and unstable steps. More relevantly, we have shown that the dimension of the feature vector can be reduced significantly, obtaining a simple self-explainable classifier. We have shown that, no matter which method is used in the dimension reduction, similar features are detected as the most relevant showing a consistency among the different feature spaces. In particular, the trunk inclination has emerged as the most relevant gait descriptor in order to analyze the robot stability. Moreover, the swing foot horizontal position appeared consistently as the second most relevant descriptor. This result agrees with previous work on biped gait stability, both for humans [22] and robots [33]. These works show that biped gait stability requires that the trunk is prevented from falling with an adequate feet placement. This is a key issue for future implementations in real robots, where many other concerns beyond the accuracy performance on the prediction must be taken into account; e.g., on the number of sensors or controller to be installed on the robot.

One limitation of this approach is that in order to model the problem as a supervised classification one, we need to run many simulations previously calculating the true class by the PSV. It requires a lot of time to run these simulations to obtain the dataset. The good thing is that we proposed a thoughtful way to produce this dataset, so that once generated, it can be used as a benchmark to train future new classifiers.

## Figures and Tables

**Table 1:** Robotic model parameters.

	Index		
	1 and 5	2 and 4	3
mass (m)	0.254 kg	0.780 kg	3.861 kg
length (l)	0.15 m	0.15 m	0.2095 m
Inertia (I)	4.34 kg·cm^2^	8.74 kg·cm^2^	141.48 kg·cm^2^
distance (c)	0.090 m	0.085 m	0.138 m
$U_{max}$	11.3 N·m		

**Table 2:** Performance metrics for each classifier.

Metric	Statistical	Temporal	Advanced	Initial
Balanced Accuracy	0.8838	0.8795	0.8790	0.7853
Precision	0.8834	0.8845	0.8828	0.7356
Recall	0.8842	0.8730	0.8740	0.8905
F1 Score	0.8838	0.8787	0.8784	0.8057
Specificity	0.8832	0.8860	0.8840	0.6800
NPV	0.8841	0.8746	0.8752	0.8613
ROC AUC	0.9526	0.9502	0.9552	0.8556
Average Precision	0.9532	0.9517	0.9569	0.8155

**Table 3:** Performance metrics for the corresponding classifier when trained with the top 5 statistical, temporal, or advanced features according to the MDI, Permutation (Perm.), Extra Trees (ET), and PCA criteria.

Metric	Statistical				Temporal				Advanced			
	**MDI**	**Perm.**	**ET**	**PCA**	**MDI**	**Perm.**	**ET**	**PCA**	**MDI**	**Perm.**	**ET**	**PCA**
Balanced Accuracy	0.8484	0.8550	0.8566	0.8006	0.8484	0.8550	0.8566	0.7863	0.8484	0.8550	0.8566	0.8014
Precision	0.8543	0.8538	0.8672	0.8141	0.8543	0.8538	0.8672	0.7921	0.8543	0.8538	0.8672	0.8011
Recall	0.8400	0.8568	0.8423	0.7793	0.8400	0.8568	0.8423	0.7763	0.8400	0.8568	0.8423	0.8018
F1 Score	0.8471	0.8553	0.8545	0.7963	0.8471	0.8553	0.8545	0.7841	0.8471	0.8553	0.8545	0.8014
Specificity	0.8568	0.8533	0.8710	0.8220	0.8568	0.8533	0.8710	0.7963	0.8568	0.8533	0.8710	0.8010
NPV	0.8426	0.8562	0.8467	0.7883	0.8426	0.8562	0.8467	0.7806	0.8426	0.8562	0.8467	0.8016
ROC AUC	0.9282	0.9316	0.9321	0.8799	0.9282	0.9316	0.9321	0.8707	0.9282	0.9316	0.9321	0.8891
Average Precision	0.9286	0.9306	0.9343	0.8867	0.9286	0.9306	0.9343	0.8771	0.9286	0.9306	0.9343	0.8910

## Data Availability

The data presented in this study are available on request from the authors.
